# Changes in Expression of DNA-Methyltransferase and Cannabinoid Receptor mRNAs in Blood Lymphocytes After Acute Cannabis Smoking

**DOI:** 10.3389/fpsyt.2022.887700

**Published:** 2022-07-04

**Authors:** Robert C. Smith, Henry Sershen, David S. Janowsky, Abel Lajtha, Matthew Grieco, Jon A. Gangoiti, Ilya Gertsman, Wynnona S. Johnson, Thomas D. Marcotte, John M. Davis

**Affiliations:** ^1^Nathan Kline Institute for Psychiatric Research, Orangeburg, NY, United States; ^2^Department of Psychiatry, NYU Grossman School of Medicine, New York University, New York, NY, United States; ^3^Department of Psychiatry, University of California, San Diego, San Diego, CA, United States; ^4^Department of Pediatrics, University of California, San Diego, San Diego, CA, United States; ^5^Department of Psychiatry, Psychiatric Institute, University of Illinois Chicago, Chicago, IL, United States

**Keywords:** cannabis, CB2 receptor mRNA, DNMT, TET, epigenetics, schizophrenia

## Abstract

**Background:**

Cannabis use is a component risk factor for the manifestation of schizophrenia. The biological effects of cannabis include effects on epigenetic systems, immunological parameters, in addition to changes in cannabinoid receptors 1 and 2, that may be associated with this risk. However, there has been limited study of the effects of smoked cannabis on these biological effects in human peripheral blood cells. We analyzed the effects of two concentrations of tetrahydrocannabinol (THC) vs. placebo in lymphocytes of a subset of participants who enrolled in a double-blind study of the effects of cannabis on driving performance (outcome not the focus of this study).

**Methods:**

Twenty four participants who regularly use cannabis participated in an experiment in which they smoked cannabis cigarettes (5.9 or 13.4% THC) or placebo (0.02%) *ad libitum*. Blood samples were drawn at baseline and several times after smoking. Lymphocytes were separated and stored at –80^°^C for further analysis. Samples were analyzed for mRNA content for cannabinoid receptors 1 (CB1) and 2 (CB2), methylation and demethylating enzymes (DNMT, TET), glucocorticoid receptor (NRC3) and immunological markers (IL1B, TNFα) by qPCR using TaqMan probes. The results were correlated with THC whole blood levels during the course of the day, as well as THCCOOH baseline levels. Statistical analyses used analysis of variance and covariance and *t*-tests, or non-parametric equivalents for those values which were not normally distributed.

**Results:**

There were no differences in background baseline characteristics of the participants except that the higher concentration THC group was older than the low concentration and placebo groups, and the low concentration THC group had higher baseline CB2 mRNA levels. Both the 5.9 and 13.4% THC groups showed increased THC blood levels that then decreased toward baseline within the first hour. However, there were no significant differences between THC blood levels between the 5.9 and 13.4% groups at any time point. At the 4-h time point after drug administration the 13.4% THC group had higher CB2 (*P* = 0.021) and DNMT3A (*P* = 0.027) mRNA levels than the placebo group. DNMT1 mRNA levels showed a trend in the same direction (*P* = 0.056). The higher 13.4% THC group had significantly increased CB2 mRNA levels than the 5.9% concentration group at several post drug administration time points and showed trends for difference in effects for between 5.9 and 13.4% THC groups for other mRNAs. TET3 mRNA levels were higher in the 13.4% THC group at 55 min post-cannabis ingestion. When the high and lower concentration THC groups were combined, none of the differences in mRNA levels from placebo remained statistically significant. Changes in THC blood levels were not related to changes in mRNA levels.

**Conclusion:**

Over the time course of this study, CB2 mRNA increased in blood lymphocytes in the high concentration THC group but were not accompanied by changes in immunological markers. The changes in DNMT and TET mRNAs suggest potential epigenetic effects of THC in human lymphocytes. Increases in DNMT methylating enzymes have been linked to some of the pathophysiological processes in schizophrenia and, therefore, should be further explored in a larger sample population, as one of the potential mechanisms linking cannabis use as a trigger for schizophrenia in vulnerable individuals. Since the two THC groups did not differ in post-smoking blood THC concentrations, the relationship between lymphocytic changes and the THC content of the cigarettes remains to be determined.

## Introduction

Delta-9-tetrahydrocannibinol (THC) is the main biologically active constituent of the cannabis plant. Although the strength of the correlation, and suggestions of causality, remain unclear, cannabis use has been associated with various adverse mental disorders, including psychosis and schizophrenia in some individuals (1, 2). A recent GWAS analyses found significant genetic correlations with mental-health-related traits, including smoking, alcohol use, schizophrenia, and risk taking. Individuals with schizophrenia have a higher risk to start using cannabis (3). First episode psychosis patients show more frequent and increased psychotic-like experiences at greater frequency of use and higher concentrations of cannabis used compared to controls (4).

The biological effects of cannabis include effects on epigenetic systems, immunological parameters, in addition to cannabinoid receptors 1 and 2. Decreased mRNA expression of CB1 receptor encoded by the CNR1 gene has been reported in the DLPFC of patients with schizophrenia (5). THC or ethanol use was associated with dysregulated expression of CNR1 in the PFC of patients with affective disorder, and the expression of CNR1 was significantly upregulated in the PFC of schizophrenia patients who completed suicide (5). Long-term use of cannabis/cannabis has been reported to alter the endocannabinoid system, including an increase in Cannabinoid Receptor (CBR) 1 and 2 messenger RNA (mRNA) in blood (PBMC) cells (6). Even after long-term abstinence, blood CB2 mRNA is still elevated (6). In another study, CB1 receptor expression levels and methylation status appeared to be altered in participants with THC dependence as measured in peripheral blood (7). In this study, CB1 mRNA was decreased in THC-dependent smokers accompanied by a higher CB1 promoter methylation associated with the reduced amount of CBR1 mRNA, whereas CB2 mRNA was not different (7). Methylation rates in two synapse genes [microtubule-associated-protein Tau (MAPT) and neurexin (NRXN1)] measured in blood were lower in non-THC-consumer schizophrenics but increased with consumption of THC (8). Inflammatory disturbances are also evident with chronic psychotic disorders and CB2 receptors are expressed on immune cells. A recent study found lower levels pro-inflammatory cytokine (IL-6) in cannabis-using patients that correlated with higher psychopathology scores (9).

To further investigate the effects of smoked marihuana (cannabis) on these biological effects in human peripleural blood cells, we analyzed its effects on DNA methylation, CB receptor and immunological mRNAs after two concentrations of cannabis (THC) vs. placebo in lymphocytes of a subset of subjects participating in a double-blind study of the effects of cannabis on driving performance.

## Materials and Methods

### Participants and Study Design

**FIGURE 1:**
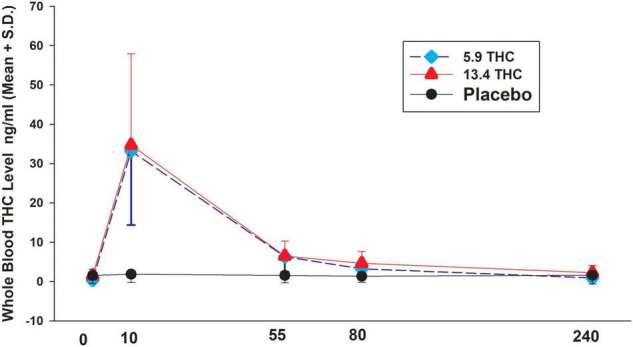
THC levels in three treatment groups. Time after smoking (minutes).

This report is sub-study chemical analysis of lymphocyte samples, derived from a double-blind randomized experimental study, conducted at the University of California San Diego Center for Medicinal Cannabis, of the effects of cannabis smoking, which involved participants smoking active cannabis containing cigarettes at two concentrations of THC (high concentration 13.4% THC, low concentration 5.9% THC) or smoking placebo (0.02%) *ad libitum* (“as you would at home to get high”). Details of the full study, sources of cannabis, inclusion and exclusion criteria, dose administration by smoking and relevant IRB approval are presented in detail in previous publications (10–12). Participants were self-reported cannabis users who were asked to abstain from using cannabis for 2 days prior to the start of this study. In this sub-study where samples of WBC and plasma were obtained there were 11 participants in the placebo group, 5 in the 5.9% THC group and 8 in the 13.4% THC group. Participants performed a simulated driving task and filled out a psychological questionnaire over the next 5 h. Driving simulator performance was evaluated *via* 25-min driving simulations prior to smoking, and again at 4 time points after smoking (12). The Composite Drive Score (CDS), is comprised of key variables from the simulations, normalized to a common metric (z scores derived from the pre-smoking drive of all 191 study participants), and represents global driving performance. Here we report the change in CDS from the pre-smoking performance to the subsequent time points. Six milliliter blood samples were taken during the study period, both before the start of the study and at several time points, during the 5-h study (see Figure 1 for approximate time points of blood draws). Two milliliters of blood were centrifuged (400 × g) with equal volume of histopaque-1077. The lymphocyte layer was aspirated off, mixed with 10 mL of phosphate buffer (PBS) and recovered by centrifugation again (550 × g) (Lymphocytes were not obtained in the 10 min THC blood sample). After centrifugation samples were stored at −80^°^C. Lymphocyte samples were transferred in dry ice to the neurochemistry section of Nathan Kline Institute for Psychiatric Research (NKI) for further analysis.

### Assays for Tetrahydrocannabinol and THCCOOH

Assays for THC and THCCOOH were performed in whole blood by LC-MS using validated previously published methods as fully described in previous publications (10, 11).

### Assays for mRNA in Lymphocytes

Samples available for analysis for mRNA values were collected at baseline, and at 55 min, 1 h 20 min, and 4 h post smoking the experimental cigarette. RNA was extracted from lymphocyte pellets with a TRIzol procedure. First strand cDNA was prepared using the Invitrogen SuperScript VILO cDNA Synthesis kit; up to 2.5 μg RNA was reacted with reagent mix, incubated at 42^°^C for 60 min, and terminated at 85^°^C for 5 min. For qPCR, TaqMan Universal PCR Master Mix was used for target amplification using the cDNA template and using primer/probes from the TaqMan Gene Expression Assay mix (see Table 1 for probes). Samples were assayed (Agilent Technologies, Stratagene Mx 3000P Multiplex Quantitative PCR System) in triplicate, normalized against β-actin as the housekeeping gene, and ddCt = 2^(−dt) values calculated. For details on qPCR assay procedures refer to our recent published paper (13).

**TABLE 1:** Taqman primer probes for THC study.

Gene symbol	Taqman gene expression assay	Gene name
ACTB	Hs01060665_g1	Actin beta Beta-Actin- actin isoform—generally considered stably expressed reference gene- house-keeping gene
CNR1 (CB1)	Hs01038522_s1	Cannabinoid receptor 1–low in peripheral cells, in cells of immune system, decrease with THC
CNR2 (CB2)	Hs05019229_s1	Cannabinoid receptor 2 -2- 3 times higher than CNR1 in peripheral tissue
IL1β	Hs01555410_m1	Interleukin 1 beta–cytokine modulates immune response- increase in THC smokers
TNFAIP8	Hs02621508_s1	TNF alpha induced protein 8–expressed in immune cells
DNMT1	Hs00154749_m1	DNA (cytosine-5-)-methyltransferase 1- regulate gene expression- add methyl group to cytosine- increased with THC
DNMT3A	Hs01027166_m1	DNA methyltransferase 3 alpha
TET1	Hs00286756_m1	Tet methylcytosine dioxygenase 1—methyl to hydroxymethyl cytosine, decreased with some drugs-cocaine
TET3	Hs00379125_m1	Tet methylcytosine dioxygenase 3
NR3C1 glucocorticoid	Hs00353740_m1	Nuclear receptor subfamily 3 group C member 1

### Psychological Effects of Cannabis

Psychoactive effects ratings of feeling “high” were obtained from participants’ responses of the Psychoactive Scale -Brief, a Likert type scale with ratings of 0–100 which is described in more detail in a previous publication (14). Ratings were obtained at 30, 90, 210, and 270 min post THC or placebo administration.

### Statistical Analysis

Statistical analysis used SPSS 25, using parametric general linear model analysis of the three drug administration groups’ values and *t*-tests for normally distributed values, and non-parametric Kruskal–Wallis analysis of variance for variables which deviated markedly from normal distribution (assessed by SPSS explore). Statistical significance was set at *P* < 0.05, two sided. The main analysis of mRNA values was on difference scores from baseline of each participant at each time point. A subsidiary analyses used age as a covariate for the mRNA difference analysis. The influence of THC levels and baseline THCCOOH concentration levels on mRNA values and on psychological high scores was explored with correlation analysis.

## Results

Participants characteristics are presented in Table 2. One subject in the 5.9% THC group was excluded from analysis because all his THC levels both at baseline and during 4 h after smoking came back “0” (we do not know whether he actually smoked the cannabis joint he received) and the analyzed n for this group is 4 participants. There were no significant differences in sex, previous frequency of cannabis use, or baseline THC for THCCOOH concentration levels in the three treatment groups, although the 5 participants who smoked the 5.9% concentration of THC, appeared to have lower baseline THC and THCCOOH concentration levels, but statistical analysis did not reveal a significant difference (*P* < 0.05) from the other two groups. However, the group who smoked 13.4% THC was significantly older than the other two groups. Most of the participants had a history of high or moderate cannabis use, but there was no difference between the three treatment groups in past history of frequency of cannabis use. There was no difference between the groups in baseline CDS driving scores.

**TABLE 2:** Characteristics of subjects in the three THC concentration groups.

Characteristic	Placebo	5.9% THC	13.4% THC	Test
	(*n* = 11)	(*n* = 3–4)	(*n* = 8)	
Age mean	27.00 ± 5.81	24.00 ± 2.58	37.38 ± 11.99	*H* = 6.172, *df* = 2, *P* = 0.046
Median	23.00	24.00^a^	31.00^a†^	
Sex (M/F) (*n*)	6/5	3/1	6/2	FET = 1.061, *P* = 0.62
Baseline mean	16.65 ± 17.03	4.90 ± 4.93	20.44 ± 20.11	*H* = 2.611, *df* = 2, *P* = 0.27
THCCOOH median level	6.10	3.10	13.20	
Baseline mean	1.53 ± 2.00	0.33 ± 0.65	1.50 ± 1.60	*H* = 1.697 *df* = 2, *P* = 0.43
THC median level	0.70	0.00	1.10	
Characterization of past Marijuana use (High, middle, low) (*n*)	3/7/1	1/2/0	2/5/1	FET = 1.176, *P* = 1.00
CDS baseline score (mean)	−0.075 ± 0.685	−0.478 ± 0.230	0.242 ± 0.518	*F* = 1.722, *df* = 2.21, *P* = 0.21

*Higher CDS scores indicate poorer driving performance. H = test statistic from Kruskal–Wallis analysis of variance. Parametric analysis of age, TCCOOH and baseline TCH levels shows similar results. F = analysis of variance test statistic. FET, fishers exact test. ^†^Difference from placebo P = 0.056. ^a^Difference between 5.9 and 13.4 group P = 0.023.*

### Tetrahydrocannabinol Levels

Both the 5.9 and 13.4% concentrations of cannabis increased THC with a peak at 10 min after smoking and then decreasing over the next 4 h with significant differences from placebo group. However, there were no statistically significant differences (*P* < 0.05) in THC levels between the 5.9 and 13.4% concentration (see Figure 1). THC levels in the placebo group remained constant at very low levels. There was a highly significant correlation of baseline THCCOOH levels and baseline THC levels (rho = 0.80 *P* < 0.001). In the groups who received active THC there was a correlation of baseline THCCOOH concentration levels and increase in THC levels at the 4-h time point after drug administration (*r* = 0.58 *P* = 0.047).

### Composite Drive Score Scores

The subjects smoking active cannabis showed significantly (*P* < 0.02) increased CDS scores (poorer driving performance) at 30 and 90 min and 3 h 30 min (*P* = 0.032) after smoking compared to placebo (see Supplementary Table 3). There were no differences in CDS effects between the 5.9 and 13.4 THC groups. There were no significant correlations between CDS change score and change in THC blood levels in active cannabis groups. These results for cannabis effects on CDS scores for the sub-sample reported in this paper are similar to the effects on cannabis reported for the full sample of 191 subjects in our recently published paper although in the full study the CDS scores were not significantly different at 3 h 30 min (12).

### Lymphocyte mRNA Levels

There were no significant differences between groups in baseline mRNA levels, except for the cannabinoid receptor 2 levels, where the 5.9% THC group had significantly higher levels compared to placebo and 13.4% THC (Table 3). When we analyzed difference in mRNA levels, at three time points post smoking, 55 min, 1 h 20 min, and 4 h (each difference time_*i*_ value-baseline values), there were significant differences in the groups who received 13.4% THC compared to the placebo group in mRNA levels in cannabinoid receptor 2 and DNMT3A at the 4-h time point (Table 4) and a difference in TET3 mRNA between the 13.4% and placebo groups at the 55-min time point. There was a strong trend for DNMT1 mRNA to also be higher than placebo in the 13.4% THC group at the 4-h time point (*P* = 0.056). There were also some striking differences in response of the 4 participants who received the 5.9% THC concentration vs. the 8 who received the 13.4% THC concentration, on some of these mRNA measures. Cannabinoid receptor 2 levels increased at the 13.4% THC concentration but decreased at the 5.9% THC concentration with significant differences from placebo at some time points. DNMT3A was increased at 4 h post smoking in the 13.4% THC concentration with a similar trend for DNMT1, but the 5.9% THC concentration showed an opposite trend. There were no significant effects on other epigenetic-related mRNAs [TET1, glucocorticoid receptor, or immunological related makers (IL1B, TNFα)]. There were trends for differences in response for these other mRNAs between the 5.9 and 13.4% concentration in opposite directions which were not statistically significant. Given that the 13.4% THC group was significantly older than the other groups age was added as a covariate in a separate analysis; the pattern of effects with the adjusted mean differences were similar to the original analysis, but some of the differences for cannabinoid receptor 2 and DNMT3A mRNAs were lower in statistical significance (Supplementary Table 1). When the 5.9 and 13.4% THC groups were combined there were no differences from placebo. There was a positive correlation of CB2 increase and TNFα increase at the 55-min time point after cannabis administration (*r* = 0.54 *P* = 0.007, *n* = 23) but not at later time points.

**TABLE 3:** Baseline mRNA levels in the three treatment groups.

mRNA	Placebo	5.9% THC	13.4% THC	Analysis
	(*N* = 11)	(*N* = 4)	(*N* = 8)	
*CB1*	8.93 ± 15.54	6.47 ± 5.59	2.45 ± 1.69	*F* = 0.771, *df* = 2.20, *P* = 0.476
*CB2*	248.77 ± 179.16	419.42 ± 262.17†^b^	148.10 ± 73.42^b^	*F* = 3.486, *df* = 2.20, *P* = 0.050
*DNMT1*	129.45 ± 109.35	69.29 ± 38.08	114.09 ± 92.16	*F* = 0.579, *df* = 2.20, *P* = 0.569
*DNMT3A*	391.44 ± 408.32	238.62 ± 160.55	293.29 ± 233.74	*F* = 0.403, *df* = 2.20, *P* = 0.674
*IL1B*	179.83 ± 186.75	228.49 ± 246.05	130.56 ± 103.51	*F* = 0.449, *df* = 2.20, *P* = 0.645
*NR3C*	406.01 ± 320.47	201.60 ± 137.64	214.37 ± 123.88	*F* = 1.852, *df* = 2.20, *P* = 0.183
*TET1*	33.09 ± 22.95	15.98 ± 10.92	18.12 ± 13.10^†^	*F* = 2.085, *df* = 2.20, *P* = 0.150
*TET3*	165.58 ± 125.90	89.15 ± 47.10	75.88 ± 57.29^†^	*F* = 2.243, *df* = 2.20, *P* = 0.132
*TNF*α	532.38 ± 480.38	366.40 ± 242.32	445.56 ± 257.26	*F* = 0.306, df = 2.20, *P* = 0.740

Each value is mean±SD (ddCt = 2^(−dt) × 10^4^). F is overall analysis for treatment effect. Difference between 5.9 and 13.4 THC vs. placebo: *P < 0.05, ^†^P < 0.10.

*Difference between 5.9 and 13.4 THC groups: ^b^P < 0.05.*

*For CB1 Placebo was non-normally distributed and non-parametric Kruskal–Wallis tests also showed no significant difference: H = 4.536, df = 2, P = 0.104.*

**TABLE 4:** Differences in mRNA levels from baseline in leucocytes in three treatment groups after smoking marijuana or placebo cigarettes.

mRNA	Time after smoking	Placebo	5.9% THC	13.4% THC	Analysis, treatment effect
		(*N* = 11)	(*N* = 4)	(*N* = 8)	
*Cannabinoid receptor 2 (CB2)*	55 min post	−40.92 ± 171.03	−216.66 ± 134.12^b^*	+64.72 ± 40.32^b^	*F* = 5.922, *df* = 2.20, *P* = 0.010
	1 h 20 min post	−55.00 ± 146.67	−219.40 ± 229.07 ^b^l	+29.22 ± 48.36^b^	*F* = 4.238, *df* = 2.20, *P* = 0.029
	4 h post	−64.65 ± 154.35	−163.89 ± 177.63^b^	+132.37 ± 186.35^b^*	*F* = 5.026, *df* = 2.20, *P* = 0.017
*DNMT1*	55 min post	+2.08 ± 40.21	+4.36 ± 23.59	+18.93 ± 40.68	*F* = 0.473, *df* = 2.20, *P* = 0.630
	1 h 20 min post	+7.30 ± 68.53	+13.63 ± 22.47	+19.31 ± 52.93	*F* = 0.099, *df* = 2.20, *P* = 0.906
	4 h post	+9.48 ± 60.85	+1.75 ± 17.92	+57.21 ± 43.75l	*F* = 2.551, *df* = 2.20, *P* = 0.103
*DNMT3A*	55 min post	−3.27 ± 169.75	−36.98 ± 109.06	+87.87 ± 182.33	*F* = 1.000, *df* = 2.20, *P* = 0.386
	1 h 20 min post	+7.64 ± 340.40	+61.14 ± 206.69	+60.37 ± 202.04	*F* = 0.102, *df* = 2.20, *P* = 0.903
	4 h post	−11.18 ± 310.57	+15.12 ± 100.00	+258.59 ± 161.83*	*F* = 3.075, *df* = 2.20, *P* = 0.068
*IL1B*	55 min post	+16.95 ± 170.83	−104.52 ± 170.75	+11.64 ± 111.70	*F* = 1.009, *df* = 2.20, *P* = 0.382
	1 h 20 min post	+1.27 ± 192.37	+7.01 ± 130.35	+52.08 ± 96.90	*F* = 0.262, *df* = 2.20, *P* = 0.772
	4 h post	+9.74 ± 201.08	−113.76 ± 233.55	+91.46 ± 103.07	*F* = 1.764, *df* = 2.20, *P* = 0.197
*NR3C*	55 min post	−7.00 ± 134.74	−29.14 ± 36.25	+78.53 ± 102.47	*F* = 1.740, *df* = 2.20, *P* = 0.201
	1 h 20 min post	+17.36 ± 280.38	+16.30 ± 71.27	+106.86 ± 112.52	*F* = 0.473, *df* = 2.20, *P* = 0.630
	4 h post	+170.89 ± 578.04	−34.09 ± 57.40	+167.20 ± 94.59	*F* = 0.401, *df* = 2.20, *P* = 0.675
*TET1*	55 min post	−2.60 ± 13.06	−4.87 ± 5.73	+3.16 ± 12.68	*F* = 0.775, *df* = 2.20, *P* = 0.474
	1 h 20 min post	+5.73 ± 25.27	−0.02 ± 8.71	+3.77 ± 9.45	*F* = 0.135, *df* = 2.20, *P* = 0.874
	4 h post	+25.56 ± 73.00	+0.52 ± 8.91	+13.54 ± 7.55	*F* = 0.369, *df* = 2.20, *P* = 0.696
*TET3*	55 min post	−9.05 ± 58.27	−12.19 ± 7.41	+45.21 ± 62.34*	*F* = 2.587, *df* = 2.20, *P* = 0.100
	1 h 20 min post	−11.81 ± 123.14	+39.34 ± 73.96	+44.63 ± 49.14	*F* = 0.932, *df* = 2.20, *P* = 0.410
	4 h post	+51.45 ± 181.07	+2.59 ± 11.65	+70.13 ± 38.81	*F* = 0.362, *df* = 2.20, *P* = 0.701
*TNF*α	55 min post dose	−10.55 ± 229.33	−132.23 ± 155.88	−3.98 ± 127.73	*F* = 0.721, *df* = 2.20, *P* = 0.499
	1 h 20 min post	−44.39 ± 349.10	−41.05 ± 177.04	+31.38 ± 227.10	*F* = 0.175, *df* = 2.20, *P* = 0.841
	4 h post dose	−12.15 ± 372.69	+17.25 ± 79.93	+202.57 ± 300.82	*F* = 1.106, *df* = 2.20, *P* = 0.350

	**Median difference**				**Kruskal–Wallis Test**

*Cannabinoid receptor 1 (CB1)*	55 min post	−0.26	−2.43	+0.93	H = 4.533, *df* = 2, *P* = 0.104
	1 h 20 min post	+0.06	−1.79	+0.15	*H* = 2.121, *df* = 2, *P* = 0.346
	4 h post	−1.98	−2.47	+1.17	*H* = 3.548, *df* = 2, *P* = 0.172

Each value is Mean ± SD (ddCt = 2^(−dt) × 10^4^), except for CB1 was not normally distributed where median is used to express central tendency. F is from analysis of variance (general linear model) from drug treatment effect. H is from Kruskal–Wallis analysis of variance for non-normative data. Difference of each THC group values from Placebo Group by LSD t-test: *P < 0.05, ^†^P < 0.10; Difference between 5.9 THC and 13.4 THC by LSD t-test. **^b^**P ≤ 0.01.

THCCOOH baseline levels and differences in THC blood levels during the study in the different treatment groups did not appear to be important factors influencing the changes in mRNA levels. There were no consistent correlations between changes in THC blood levels and changes in mRNA levels. There were no significant correlations between baseline THCCOOH concentration levels changes in any of the mRNA’s (cannabinoid receptor 2, DNMT3A, DNMT1, TET3) which showed significantly differences or strong trends for difference between the three treatment groups. There were also no significant correlations between baseline THCCOOH concentration and baseline mRNA levels.

There were no significant correlations between CDS change scores and change in mRNA levels except for one correlation in the 13.4 THC group between CDS change 3 h 30 min after smoking and DNMT3A change at 1 h 20 min after smoking (*r* = −0.78 *p* = 0.023 *n* = 8) whose substantive meaning is unclear.

The degree participants experienced feeling associated with a “high” after smoking placebo at 2 different cannabis concentrations showed that there was a significant difference in scores between the cannabis and placebo groups but not between the two cannabis concentration groups (Supplementary Table 2). Although the two cannabis groups did not significantly differ, the 13.4% had a trend for higher scores than the 5.9% group at the early assessments time points. There were significant correlations of high rating with THC blood level increase; sum of high ratings over the course of the experiment correlated with blood level increase from baseline at 10 min (*r* = + 0.641 *P* = 0.001) and with blood levels at 55 min (*r* = + 0.485 *P* = 0.019) and 80 min (*r* = + 0.519 *P* = 0.011); and there were several correlations between high ratings at 30 and 90 min with some of the THC blood level increase from baseline at one or more time points. There were a few correlations between high scores and mRNA changes. Total sum of high ratings was correlated at the 4-h time point in the 13.4% concentration group only with NR3C change (*r* = + 0.735, *P* = 0.038, *n* = 8) and IL1b change (*r* = + 0.788, *P* = 0.020 *n* = 8). In the total sample High score at 1 h 30 min time point was correlated with the change in cannabinoid receptor 2 change at 4 h (*r* = −0.469, *P* = 0.024, *n* = 23).

## Discussion

In this experiment acute smoking of higher concentration of THC modulated an increase in cannabinoid receptor CB2 and methylating enzymes DNMT3a and DNMT1 mRNAs at 4 h after ingestion when the blood levels of THC were very low. Of note, though, the high and low THC concentration groups showed similar blood THC levels, and the changes were not related to THC blood levels either at peak levels (10 min after ingestion) or other time points. Previous reports from this research (10, 11) suggested that the smokers self-titrated the concentration of cannabis consumed to achieve their desired level of highness, which likely accounts for the similar THC levels in the 5.9 and 13.4% concentration groups.

The finding that there was a marked difference in the effects of the 5.9% vs. the 13.4% THC concentration, and that changes in mRNAs were not correlated with blood levels suggest a different independent mechanism of THC or other components in the smoked cannabis cigarettes producing the changes in the high concentration group. We cannot be certain whether the very small sample size of the 5.9% concentration group and its higher levels of CB2 at baseline influence the marked difference between effects of the 5.9 and 13.4% concentration. However, there were no significant differences in baseline values of other mRNAs between the three test groups, and the results for these other mRNAs also tended to show a difference in effects between the 5.9 and 13.4% concentration groups at one or more time points. Although CB2 receptors are thought to be involved in immunomodulatory responses (15, 16), and previous data has suggested that they may reduce levels of cytokines such as TNFα (17) and IL1B, we did not find a significant change in these mRNAs, and there were no significant negative correlations between mRNA levels of CB2 and TNFα or IL1B. This suggests that the act of smoking of a THC cigarette in previous cannabis users does not have acute short-term effects on this immune response expressed in lymphocytes.

Increased mRNA levels of DNMT with the high dose of cannabis that we found have not been previously reported in the lymphocytes of cannabis users or associated with acute THC administration. The only other human peripheral cell research involving DNMT we discovered was a report of decreased methylation of DNMT3b receptors and decreased DNMT3b in human follicular cells in cannabis exposed women (18). Seminal THC has been found to be correlated with serum THC and THC metabolites (19) and many aspects of the human male reproduction can be modulated by cannabis (20) possibly *via* DNMT. DNA methylation plays critically important functions during spermatogenesis in mammals that is catalyzed by DNA methyltransferase (DNMT) enzymes, and is related to male infertility (21). The antidepressant effects of cannabidiol (CBD), a non-psychotomimetic component of *Cannabis sativa* plant was suggested to involve regulation of stress-induced changes in DNA methylation in mouse brain (22). We have previously reported that increased DNMT1 and DNMT3a mRNAs are found in the lymphocytes and post-mortem brain samples from schizophrenics compared to non-psychotic controls, and therefore may be a potential biomarker contributing to this illness (13). Higher DNMT levels could result in hypermethylation of the GAD67 promoter and consequently lead to lower synthesis of brain GABA which has been implicated as one of the biological mechanisms underlying schizophrenia (23). There is strong evidence from both epidemiological studies and studies of acute administration of THC, that cannabis consumption especially in adolescent years is associated with increased risk of early onset schizophrenia and symptoms associated with schizophrenia in vulnerable individuals (24–28). Multiple studies also show that up to 45% of normals can have increases in psychotic symptoms measured on the PANSS scale after cannabis administration, but the psychotic like effects of cannabis in schizophrenics are more pronounced than in normal controls (29, 30). Pre-clinical studies in rats have found that adolescent exposure to THC led to persistent attenuation of GABAergic function combined with disruptions of cortical gamma oscillatory activity in pre-frontal cortex neurons which resulted in associated hyperactive dopaminergic activity (31, 32). Changes in the expression of cannabinoid CB1 and dopamine D2 genes is shown in schizophrenic subjects, similarly to those described in neurodevelopmental animal models (33–35). Other studies show that CB1 receptors in the brain are located in several brain regions including pre-frontal cortex and activation of CB1 receptors by THC, inhibits the release of GABA by cholecystokinin basket cells, with consequent decreased GABAergic influence on dopaminergic modulation (28). The cannabinoid CB1 receptors, as the target of THC, are present at very high levels on inhibitory (GABAergic interneurons) and at a lesser extent on excitatory (glutamatergic) terminals (36), as well as on neurons expressing dopamine D1 receptors, playing a specific role in the repertoire of different emotional behaviors included social and cognitive activity, which are affected in psychiatric disorders (37–40). Thus, it cannot be excluded that the different effect of cannabis on schizophrenia symptoms could be also due to the specific targeting the CB1 receptors expressed on different neuronal subpopulations. These lines of evidence suggest that if the increased DMNTs mRNA in lymphocytes after cannabis ingestion is also reflected in the brain, these effects could be one factor contributing to the effects of cannabis on provoking schizophrenic symptoms in individuals with increased genetic or environmental vulnerabilities to developing this illness. The higher methylation reported for MAPT and NRXN1 after THC consumption was also interpreted as possibly disadvantageous in schizophrenia, as higher methylation generally leads to reduced readability of genes, which might further impair reduced synaptic connections (8).

### Study Limitations

The main limitations of our study are the small number of participants we could assess for mRNA changes in lymphocytes. Since there was no difference in blood THC concentrations between the two THC groups, nor a linear concentration-response relationship between effects in the 5.9% vs. 13.4% THC group on mRNAs the mechanisms for the effects seen in this study remain unclear and need to be replicated with much larger samples.

## Data Availability Statement

The raw data supporting the conclusions of this article will be made available by the authors, without undue reservation.

## Ethics Statement

The studies involving human participants were reviewed and approved by the University of California San Diego Institutional Review Board (IRB 160641). The patients/participants provided their written informed consent to participate in this study. mRNA analysis of samples in this manuscript was approved by NKI IRB.

## Author Contributions

JD and TM designed the initial study and transfer of lymphocyte samples to NKI. HS, RS, and MG performed the assays and statistically analyzed assay results. RS, HS, JD, TM, and AL wrote the manuscript and contributed to revisions of the manuscript. IG and WJ arranged for drawing and processing of the blood samples. All authors contributed to the article and approved the submitted version.

## Conflict of Interest

The authors declare that the research was conducted in the absence of any commercial or financial relationships that could be construed as a potential conflict of interest.

## Publisher’s Note

All claims expressed in this article are solely those of the authors and do not necessarily represent those of their affiliated organizations, or those of the publisher, the editors and the reviewers. Any product that may be evaluated in this article, or claim that may be made by its manufacturer, is not guaranteed or endorsed by the publisher.
