# Supplementary material for: Changes in Expression of DNA-Methyltransferase and Cannabinoid Receptor mRNAs in Blood Lymphocytes After Acute Cannabis Smoking
Source: Front Psychiatry. 2022 Jul 4;13:887700. doi: 10.3389/fpsyt.2022.887700 (PMC9290435; doi:10.3389/fpsyt.2022.887700)
Supplement: Supplementary file 1 [file Data_Sheet_1.docx]

**Supplementary Data**

**Table S1** **Difference Of Post Ingestion Means From Pre-Drug Baseline With Age Adjustment** (Mean ± Std. Errror)

| mRNA | time | placebo | 5.9% THC | 13.4% THC | Anov |
| --- | --- | --- | --- | --- | --- |
| Cannabinoid receptor 2 | 2B- 55 min post dose | -32.837 ± 42.374 | -200.709 ± 71.300 **^a^*** | +45.623 ± 54.836 **^a^** | F=3.411, df=2,19, P=0.054 |
|  | 3A- 1 hr 20 min post dose | -47.340 ± 44.278 | -204.288 ± 74.503 **^a^**ƚ | +11.128 ± 57.300 **^a^** | F=2.476, df=2,19, P=0.111 |
|  | 4B- 4 hours post dose | -61.874 ± 54.424 | -158.420 ± 91.575 **^a^** | +125.827 ± 70.429 **^a^**ƚ | F=2.950, df=2,19, P=0.077 |
| DNMT1 | 2B- 55 min post dose | +6.500 ± 11.714 | +13.075 ± 19.710 | +8.497 ± 15.158 | F=0.045, df=2,19, P=0.956 |
|  | 3A- 1 hr 20 min post dose | +8.935 ± 18.678 | +16.861 ± 31.428 | +15.444 ± 24.171 | F=0.038, df=2,19, P=0.963 |
|  | 4B- 4 hours post dose | +7.569 ± 16.188 | -2.027 ± 27.239 | +61.732 ± 20.949ƚ | F=2.134, df=2,19, P=0.146 |
| DNMT3A | 2B- 55 min post dose | +16.738 ± 50.719 | +2.472 ± 85.342 | +40.642 ± 65.636 | F=0.059, df=2,19, P=0.943 |
|  | 3A- 1 hr 20 min post dose | +24.166 ± 88.889 | +93.737 ± 149.567 | +21.343 ± 115.031 | F=0.092, df=2,19, P=0.912 |
|  | 4B- 4 hours post dose | -3.360 ± 77.612 | +30.543 ± 130.592 | +240.125 ± 100.438ƚ | F=1.646, df=2,19, P=0.219 |
| IL1B | 2B- 55 min post dose | +4.943 ± 47.937 | -128.190 ± 80.660 | +39.986 ± 62.035 | F=1.369, df=2,19, P=0.278 |
|  | 3A- 1 hr 20 min post dose | -12.214 ± 48.722 | -19.587 ± 81.981 | +83.923 ± 63.051 | F=0.701, df=2,19, P=0.509 |
|  | 4B- 4 hours post dose | -7.777 ± 55.526 | -148.303 ± 93.430 **^a^** | +132.814 ± 71.856 **^a^** | F=2.496, df=2,19, P=0.109 |
| NR3C | 2B- 55 min post dose | -3.867 ± 36.431 | -22.971 ± 61.300 | +71.141 ± 47.145 | F=0.851, df=2,19, P=0.443 |
|  | 3A- 1 hr 20 min post dose | +6.645 ± 67.089 | -4.826 ± 112.886 | +132.158 ± 86.820 | F=0.635, df=2,19, P=0.541 |
|  | 4B- 4 hours post dose | +174.697 ± 132.584 | -26.585 ± 223.090 | +158.207 ± 171.577 | F=0.330, df=2,19, P=0.723 |
| TET1 | 2B- 55 min post dose | -0.710 ± 3.528 | -1.132 ± 5.936 | -1.307 ± 4.565 | F=0.006, df=2,19, P=0.994 |
|  | 3A- 1 hr 20 min post dose | +5.813 ± 6.107 | +0.148 ± 10.276 | +3.571 ± 7.903 | F=0.127, df=2,19, P=0.881 |
|  | 4B- 4 hours post dose | +27.249 ± 16.606 | +3.846 ± 27.941 | +9.563 ± 21.490 | F=0.388, df=2,19, P=0.684 |
| TET3 | 2B- 55 min post dose | -9.385 ± 17.773 | -12.855 ± 29.905 | +46.003 ± 22.999ƚ | F=1.736, df=2,19, P=0.203 |
|  | 3A- 1 hr 20 min post dose | -15.478 ± 30.709 | +32.104 ± 51.672 | +53.290 ± 39.740 | F=0.990, df=2,19, P=0.390 |
|  | 4B- 4 hours post dose | +54.095 ± 41.719 | +7.798 ± 70.198 | +63.897 ± 53.989 | F=0.209, df=2,19, P=0.813 |
| TNFAIP8 | 2B- 55 min post dose | +16.804 ± 55.876 | -78.303 ± 94.018 | -68.555 ± 72.308 | F=0.656, df=2,19, P=0.530 |
|  | 3A- 1 hr 20 min post dose | -29.765 ± 92.012 | -12.206 ± 154.821 | -3.150 ± 119.072 | F=0.016, df=2,19, P=0.984 |
|  | 4B- 4 hours post dose | +28.423 ± 96.464 | +97.260 ± 162.312 | +106.775 ± 124.833 | F=0.150, df=2,19, P=0.861 |
| Cannabinoid receptor 1 | 2B- 55 min post dose | -5.593 ± 3.963 | -4.198 ± 6.438 | +1.504 ± 4.869 | F=0.575, df=2,18, P=0.573 |
|  | 3A- 1 hr 20 min post dose | -3.992 ± 4.103 | -4.801 ± 6.665 | +1.492 ± 5.041 | F=0.363, df=2,18, P=0.700 |
|  | 4B- 4 hours post dose | +8.397 ± 25.108 | +28.098 ± 40.791 | +16.360 ± 30.851 | F=0.096, df=2,18, P=0.909 |

Difference of each THC group value from Placebo Group by LSD t-test: *=P<.05, Ϯ=P<.10; Difference between 5.9% THC and

13.4% THC by LSD t-test: **^a^**=P<.05, **^b^**=P<.01

**Table S2** **Rating on Feeling High Scale in Three Treatment Groups**

| High Score and Time | Placebo (N=11) | 5.9% THC (N=4) | 13.4% THC (N=8) | Analysis |
| --- | --- | --- | --- | --- |
| High Score at 30 minutes | 20.73 ± 21.65 | \| 62.50 ± 12.97^**^ \|  \| \| --- \| --- \| | 58.88 ± 27.34^**^ | F^SR^=8.378, df=2,20, P=0.002 |
| High Score at 90 minutes | 5.91 ± 7.11 | 16.50 ± 20.03 | 32.75 ± 24.12^**^ | F^SR^= 7.707, df=2,20, P=0.003 |
| High Score Sum  (30-270 minutes) | 33.55 ± 27.65 | 83.00 ± 29.47^*^ | 104.00 ± 55.12^**^ | F= 7.706, df=2,20, P=0.003 |

Each number represents mean ± S.D. F^SR^= analysis performed on normalized (square root) transformed values.

*, ** significantly different from placebo group *P<.05, **P<.01. The high scores assessed at 210 and 270 minutes

Did not show significant difference between the cannabis groups and the placebo group at these specific time points.

**Table S3 Change in Composite Drive Score (CDS) at Time Points After Smoking THC or Placebo Cigarettes**

| Time after Smoking | Placebo group | THC groups | T-Test |
| --- | --- | --- | --- |
| 30 minutes | -0.265 ± 0.633 | 0.462 ±0 .657 | T=2.644, df=20, P=.016 |
| 90 minutes | -0.008 ± .590 | 0.577 ± 0.438 | T=2.641, df=20, P=.016 |
| 3 hr 30 min | -0.302 ± 0.683 | 0.228 ± 0.338 | T=2.307, df=20 P=.032 |
| 4 hr 30 min | 0.044 ± 0.884 | 0.246 ± 0.546 | T=-.649, df=20, P=.54 |

Each number presents mean ± S.D. Higher CDS scores are associated with more severe driving impairment.
